# A specific microRNA profile as predictive biomarker for systemic treatment in patients with metastatic colorectal cancer

**DOI:** 10.1002/cam4.3371

**Published:** 2020-08-30

**Authors:** Dennis Poel, Elske C. Gootjes, Lotte Bakkerus, Wim Trypsteen, Henk Dekker, Hans J. van der Vliet, Nicole C. T. van Grieken, Cornelis Verhoef, Tineke E. Buffart, Henk M. W. Verheul

**Affiliations:** ^1^ Department of Medical Oncology Cancer Center Amsterdam Amsterdam UMC VU Universiteit Medical Center Amsterdam Amsterdam the Netherlands; ^2^ Department of Medical Oncology Radboud University Medical Center Nijmegen the Netherlands; ^3^ Department of Internal Medicine and Pediatrics Faculty of Medicine and Health Sciences HIV Cure Research Center Ghent University and Ghent University Hospital Ghent Belgium; ^4^ Department of Pathology Cancer Center Amsterdam Amsterdam UMC VU Universiteit Medical Center Amsterdam Amsterdam the Netherlands; ^5^ Division of Surgical Oncology Department of Surgery Erasmus MC Cancer Institute Rotterdam the Netherlands; ^6^ Department of Gastrointestinal Oncology Antoni van Leeuwenhoek Amsterdam the Netherlands

**Keywords:** chemotherapy, colorectal cancer, metastases, miRNA, response prediction, serum

## Abstract

**Background:**

Palliative systemic therapy is currently standard of care for patients with extensive metastatic colorectal cancer (mCRC). A biomarker predicting chemotherapy benefit which prevents toxicity from ineffective treatment is urgently needed. Therefore, a previously developed tissue‐derived microRNA profile to predict clinical benefit from chemotherapy was evaluated in tissue biopsies and serum from patients with mCRC.

**Methods:**

Samples were prospectively collected from patients (N = 132) who were treated with capecitabine or 5‐FU/LV with oxaliplatin ± bevacizumab. Response evaluation was performed according to RECIST 1.1 after three or four cycles, respectively. Baseline tissue and serum miRNAs expression levels of miR‐17‐5p, miR‐20a‐5p, miR‐30a‐5p, miR‐92a‐3p, miR‐92b‐3p, and miR‐98‐5p were quantified with RT‐qPCR and droplet digital PCR, respectively. Combined predictive performance of selected variables was tested using logistic regression analysis.

**Results:**

From 132 patients, 81 fresh frozen tissue biopsies from metastases and 93 serum samples were available. Based on expression levels of miRNAs in tissue, progressive disease could be predicted with an AUC of 0.85 (95% CI:0.72‐0.91) and response could be predicted with an AUC of 0.70 (95% CI:0.56‐0.80). This did not outperform clinical parameters alone (respectively *P* = .14 and *P* = .27). Expression levels of miR‐92a‐3p and miR‐98‐5p in serum significantly improved the predictive value of clinical parameters for response to chemotherapy (AUC 0.74, 95% CI:0.64‐0.84, *P* = .003) in this cohort.

**Conclusions:**

The additive predictive value to clinical parameters of the tissue‐derived six miRNA profile for clinical benefit could not be validated in patients with mCRC treated with first‐line systemic therapy. Although miR‐92a‐3p and miR‐98‐5p serum levels improved the predictive value of clinical parameters, it remained insufficient for clinical decision‐making.

## Graphical abstract

For miR‐17‐5p, miR‐20a‐5p, miR‐30a‐5p, miR‐92a‐3p, miR‐92b‐3p and miR‐98‐5p no correlation was observed between tissue and serum miRNA expression levels in patients with metastatic colorectal cancer (mCRC). The predictive value of this tissue‐derived 6 miRNA profile for clinical benefit from systemic treatment could not be validated in patients with mCRC starting first line systemic therapy.

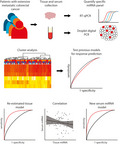

## 1 INTRODUCTION

MicroRNAs (miRNAs) are small noncoding RNAs that have an impact on many important biological processes by regulation of protein expression levels. In addition, miRNAs have favorable biomarker characteristics as they are easy to detect and resistant to degradation. Prior studies showed that tissue miRNAs are differentially expressed between normal and tumor tissue and between tumor subtypes. Furthermore, it has been observed that miRNAs from tumor cells are secreted into the circulation and can be detected in blood plasma and serum.^1^, ^2^

Since it is possible to reliably detect miRNAs in human blood specimens, interest has shifted toward using miRNAs as a liquid biomarker. Tumor specific miRNAs may be released into the blood circulation through active secretion in exosomes or through passive secretion by cell death.^3^ Patients with high tumor load are expected to have high levels of tumor specific circulating miRNAs (ci‐miRNAs). Previous studies have shown significant correlations between ci‐miRNAs and tumor stage and between paired tissue and serum miRNA expression levels.^4^, ^5^ These ci‐miRNAs could function as a minimally invasive predictive biomarker for disease monitoring upon treatment.

Among cancer types, colorectal cancer (CRC) ranks in the top 3 for incidence and mortality worldwide.^6^, ^7^ Approximately half of all patients diagnosed with CRC will have metastasis at diagnosis or develop them during the course of their disease.^8^ For patients with extensive metastatic disease, no treatment with curative intent is available. Standard of care for these patients is palliative systemic treatment that usually consists of 5‐FU‐based combination chemotherapy with oxaliplatin and/or irinotecan, anti‐VEGF targeted therapy, and anti‐EGFR antibodies. Overall survival with these modern regimens is an estimated 30 months.^9^, ^10^ Currently, only for the use of anti‐EGFR therapy a biomarker of response is available and it is recommended to exclude RAS and BRAF mutant tumors and right‐sided primary tumors from anti‐EGFR therapy.^11^, ^12^ Since approximately 10%–20% of the patients do not benefit from first‐line chemotherapy and will have progressive disease at first evaluation, there is a strong clinical need for a predictive biomarker.^9^, ^10^, ^13^, ^14^

Previously, a model to predict response to palliative systemic treatment in patients with mCRC based on primary tumor expression of miRNAs and clinicopathological factors was developed. Six miRNAs (miR‐17‐5p, miR‐20a‐5p, miR‐30a‐5p, miR‐92a‐3p, miR‐92b‐3p and miR‐98‐5p) combined with four clinical parameters (the use of adjuvant chemotherapy after resection of the primary tumor, age, tumor differentiation, and type of systemic treatment, ie, oxaliplatin or irinotecan based) were predictive for clinical benefit with an AUC of 0.78.^15^ Furthermore, expression profiles of primary CRC tissues and metastatic lesions are highly comparable.^16^

Three of these miRNAs (miR‐17‐5p, miR‐20a‐5p, and miR‐92a‐3p) belong to the miR‐17‐92 cluster, also known as oncomir‐1. miRNAs from this cluster are involved in tumor angiogenesis, treatment resistance, and CRC progression.^17^, ^18^, ^19^, ^20^ The other miRNAs, miR‐30a‐5p, miR‐92b‐3p, and miR‐98‐5p are involved in proliferation of CRC cells.^21^, ^22^, ^23^ In addition, high expression of miR‐92b‐3p has previously been related to a prolonged progression‐free survival in patients with mCRC treated with combined FOLFOX and bevacizumab.^24^ The current study aimed to prospectively validate the predictive value of clinical benefit from systemic treatment of the previously identified miRNA signature in a cohort of patients with extensive mCRC, starting first‐line palliative systemic therapy.

## 2 MATERIAL AND METHODS

### 2.1 Tissue and serum samples

In total 132 consecutive patients enrolled in a clinical trial (the ORCHESTRA trial) between May 2013 and February 2017 were included for miRNA analysis. This trial is a randomized multicenter clinical trial for patients with multiorgan colorectal cancer metastases comparing the combination of chemotherapy and maximal tumor debulking vs chemotherapy alone (clinicaltrials.gov Identifier: NCT01792934).^25^ Of all patients, tissue samples and serum biopsies were prospectively collected as part of the translational study program.

Written informed consent was obtained from all patients included in this trial.^26^, ^27^, ^28^ At baseline, one to four fresh frozen 14‐18 G needle biopsies were obtained (ultrasound or CT guided) from a metastatic lesion or from the primary tumor (endoscopically). The biopsy specimen was transferred to a storage tin and snap frozen in liquid nitrogen and subsequently stored at −80°C.

Serum samples were collected in a BD vacutainer^®^ tube, incubated upright for 1 hour and centrifuged at 1500 × *g* for 10 minutes at room temperature. Samples were aliquoted and stored at −80°C until RNA isolation. Specimens were collected prior to the start of systemic therapy.

Clinical parameters were documented and included age, gender, primary tumor sidedness (left or right), number of organs involved in metastatic disease, primary tumor in situ (yes or no), number of metastatic lesions (<5, 5‐10, >10 or diffuse disease), differentiation (well/moderately, poor), prior (neo‐) adjuvant chemotherapy (yes or no), CEA (elevated > 5 ng/µL, normal), LDH (elevated > 250 ng/µL, or normal).

All patients were treated with 5‐FU/LV or capecitabine and oxaliplatin (46‐hour continuous infusion of 5‐FU/LV and oxaliplatin in a biweekly schedule (FOLFOX) or oxaliplatin IV followed by 14 days of oral capecitabine in a 3‐week cycle (CAPOX)) ± bevacizumab at physician discretion. After three cycles of CAPOX(B) or four cycles of FOLFOX(B) a CT scan of thorax and abdomen was performed. Follow‐up scans were performed at least every 3 months and evaluated according to RECIST v 1.1.^29^

### 2.2 RNA extraction

#### 2.2.1 Tissue

Dissection of fresh frozen biopsies was performed at approximately −25°C in a cryotome. Biopsies were enriched for tumor cells by macro dissection. Twenty micrometer sections were cut and snap frozen in liquid nitrogen and stored at −80°C until RNA extraction. Multiple 5 µmol/L sections were stained with hematoxylin and eosin (H&E) to confirm the presence of tumor cells across the biopsy. As stromal infiltration is a characteristic for worse prognosis, stromal percentage was included into tumor purity estimation.^30^ The percentage of tumor field was defined as the sum of the percentage of tumor cells and the percentage of stromal cells. Samples were included if the percentage of tumor field was ≥ 30% as scored by a pathologist. RNA was isolated using the AllPrep^®^ DNA/RNA/miRNA Universal Kit (Qiagen) according to the manufacturers protocol, eluted in 30‐µL‐nuclease free water and quantified using a Nanodrop 2000 (Thermo Scientific).

#### 2.2.2 Serum

Serum RNA was extracted using the miRNeasy Serum/Plasma advanced Kit (Qiagen, former Exiqon).^31^ During RNA extraction a DNase step was included using the RNase‐Free DNase Set (Qiagen) according to the manufacturers protocol.

### 2.3 Hemolytic index

The hemolytic index (HI) of serum samples was measured using the automated Roche Modular Cobas 8000 platform according to the instructions of the manufacturer. Serum samples with a HI ≥ 10 were excluded for further analysis, since blood cells contain a lot of miRNAs and significant correlations between the HI and expression of certain ci‐miRNAs was demonstrated before.^31^, ^32^

### 2.4 miRNA quantification

#### 2.4.1 Tissue

miRNAs miR‐16‐5p, miR‐17‐5p, miR‐20a‐5p, miR‐30a‐5p, miR‐92a‐3p, miR‐92b‐3p, and miR‐98‐5p were quantified with reverse transcription quantitative PCR (RT‐qPCR) and analyzed as previously described.^15^ The chemicals from Exiqon were replaced with the chemicals from Qiagen as Exiqon merged with Qiagen, using the miRCURY LNA RT kit for cDNA synthesis and the miRCURY LNA miRNA PCR assays together with miRCURY LNA SYBR® Green for miRNA quantification (Qiagen). Cq values were normalized with miR‐16‐5p as reported previously.^4^, ^15^, ^33^, ^34^

#### 2.4.2 Serum

cDNA synthesis was performed with the miRCURY LNA RT kit (Qiagen) using 3‐µL‐extracted RNA. Serum RNA quality was assessed by measuring synthetic miRNA spike‐in of cel‐miR‐39‐3p with RT‐qPCR as described above using cel‐miR‐39‐3p miRCURY LNA miRNA PCR assay (Qiagen). Samples were selected for droplet digital PCR (ddPCR) analysis if the raw Cq value was ≤ 30.^31^ cDNA was diluted 1:40 for ddPCR. Each ddPCR reaction consisted of 9.9‐µL‐diluted cDNA, 11 µL of QX200 EvaGreen ddPCR Supermix (Bio‐Rad, Veenendaal, The Netherlands), and 1.1‐µL‐optimal concentration miRCURY LNA miRNA PCR assay. The optimal PCR assay volume was experimentally assessed for each assay using 1.0‐µL, 0.5‐µL, and if necessary 0.25‐µL assay diluted in nuclease free H_2_O (Figure S1A). A gradient (52°C‐62°C) ddPCR was performed for each assay to define the optimal annealing temperature (Figure S1B). Similar PCR assays were used as described in the section tissue miRNA quantification.

Twenty‐microliter ddPCR reaction mix and subsequently 70 µL QX200^TM^ droplet generation oil for EvaGreen (Bio‐Rad) was loaded on a DG8^TM^ Cartridge for QX200^TM^/QX100^TM^ droplet generator. Droplets were generated in a QX200^TM^ droplet generator and a total of 40 µL was loaded in a ddPCR^TM^ 96‐Well‐PCR plate (Bio‐Rad). Each plate included a nontemplate control (NTC) and positive control for each PCR assay with H_2_O and RNA from cancer cell lines as described previously.^15^ The PCR reaction was performed in a Thermal cycler (Bio‐Rad) with a three‐step protocol; (a) Enzyme activation: 5 minutes at 95°C, (b) PCR: 30 seconds at 95°C and 1 minute at primer specific annealing temperature repeated 40 times, (c) droplet stabilization: 5 minutes 4°C, 5 minutes 95°C, and at 4°C until droplet reading. Droplets were quantified in a Droplet Reader QX200^TM^ (Bio‐Rad) and the number of formed droplets was obtained using QuantaSoft software (Bio‐Rad). If less than 10 000 droplets were formed, the PCR reaction for that particular sample was repeated. Threshold estimation to define positive and negative droplets together with absolute miRNA quantification was performed using the R‐package “ddpcRquant” designed by Trypsteen and colleagues.^35^ The standard “ddpcRquant” package was designed for single plate analysis. Because the number of samples in this study exceeded the number wells of a single 96‐well plate, the script was adapted to analyze multiple plates for the same assay. The final miRNA concentration in copies per µL cDNA was log_2_ transformed and normalized for technical variation with log_2_ transformed cel‐miR‐39‐3p levels as described previously.^31^, ^33^

### 2.5 Statistical analysis

Predicting response after three to four cycles of chemotherapy was tested for two scenarios (a) clinical benefit (CB) defined as complete response (CR), partial response (PR), and stable disease (SD) together vs progressive disease (PD) and (b) CR or PR vs SD or PD. The models for response prediction as designed previously ^15^ were tested with the combined miRNA expression and clinicopathological data collected from the current cohort (FF cohort). New prediction models were formed for different clinical endpoints with multivariate logistic regression analysis using Akaike's information criterion (AIC)‐based backward selection for selection of the best model. For the new prediction models the clinical parameter “type of systemic treatment” was excluded because all patients in the current FF cohort received the same systemic treatment. Performance of the models was evaluated by comparing AUCs of paired ROC curves (clinicopathological factors vs added value of the miRNA expression to these factors) as described before.^15^

As the prediction coefficients of the miRNAs from the models in the previous study (original FFPE cohort) are based on its miRNA expression levels, the miRNA expression levels in the FF cohort were compared with the expression levels in the original FFPE cohort.^15^ Because the FF cohort consisted only of patients with stage IV disease, and the original FFPE cohort consisted of patients with stage I to IV disease, the miRNA expression data from the original FFPE cohort were grouped in stage I‐III and IV disease. Two comparisons were performed: (a) miRNA expression levels between stage I‐III and IV in the original FFPE cohort and (b) miRNA expression between stage IV in the original FFPE cohort and stage IV in the FF cohort. The Welch's two sample t test or two‐sample *t* test were used for these comparisons.

Baseline characteristics from the patients in the FF cohort were compared to the baseline characteristics from the patients in the original FFPE cohort,^15^ as well as for the patients from the serum and tissue cohorts in the FF cohort. Fisher's exact two‐sided test, Pearson two‐sided Chi‐Square test or an unpaired t test were used to test significance in baseline characteristics between the different cohorts. *P* values < .05 were considered statistically significant. Log_2_ normalized expression data were used to correlate serum vs tissue miRNA expression data. Unsupervised cluster analysis was performed with log_2_ transformed normalized serum and tissue miRNA expression levels using ward.D2 clustering and Manhattan distance. Statistical analysis was performed using the R‐package “CompareGroups” using R studio (version 1.1.423) with R software (version 3.5.0) downloaded from Bioconductor.^36^, ^37^, ^38^

## 3 RESULTS

From May 2013 to March 2017, 132 patients with mCRC were included in the trial and available for analysis. From 29 patients no baseline tissue biopsy was available and from 12 patients, no baseline serum sample was available, leaving a total of 103 tissue samples (fresh frozen cohort) and 120 serum biopsies. Both serum and tumor biopsies were available in 95 patients.

**FIGURE 1:**
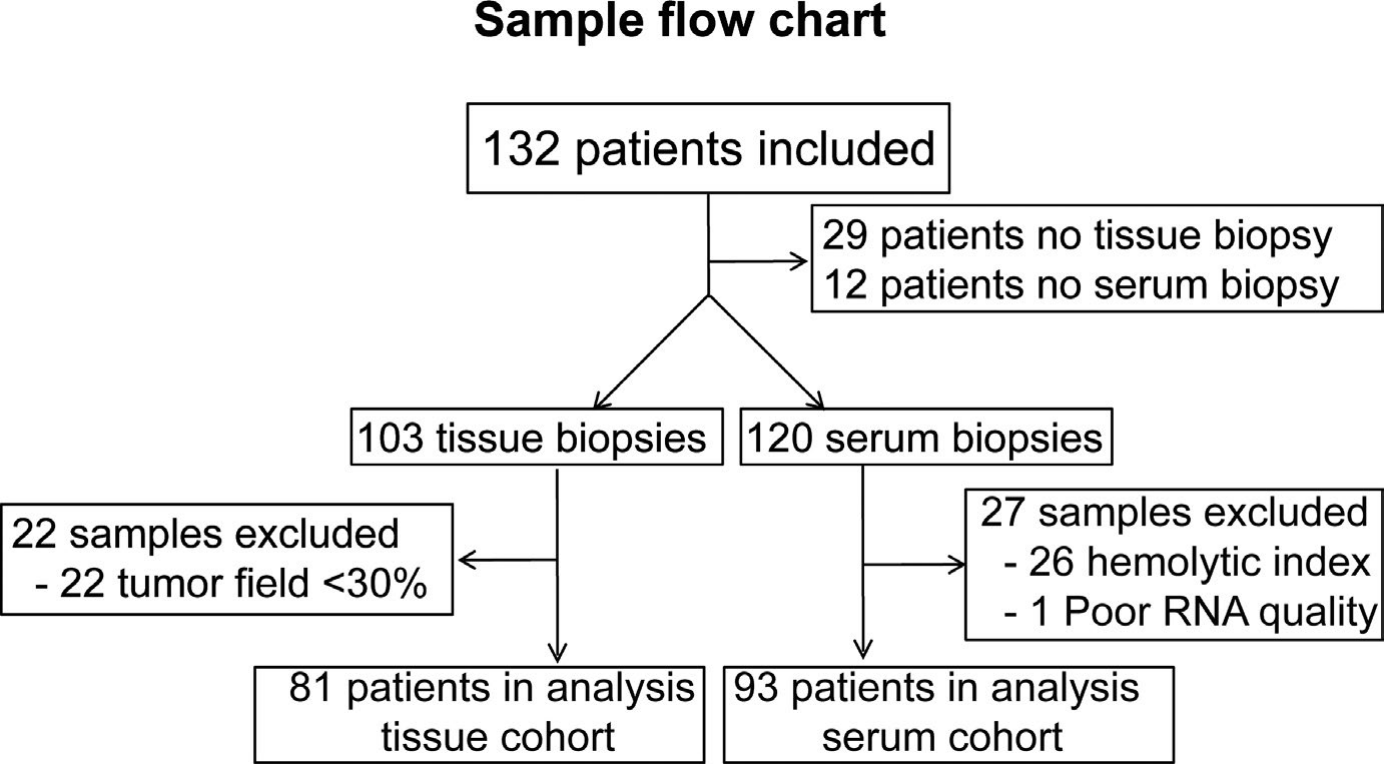
Sample flow chart of the included tissue and serum biopsies

From the available tissue samples, 22 were excluded due to a tumor field percentage < 30%, leaving a tissue cohort of 81 samples for further analyses. From 120 available serum samples, 24 were excluded due to a hemolytic index > 10, two were excluded because the hemolytic index was not assessable and one was excluded because of poor RNA quality (Figure 1), leaving a serum cohort of 93 samples. From 58 patients, miRNA expression could be quantified with good quality from both serum and tumor sample.

**FIGURE 2:**
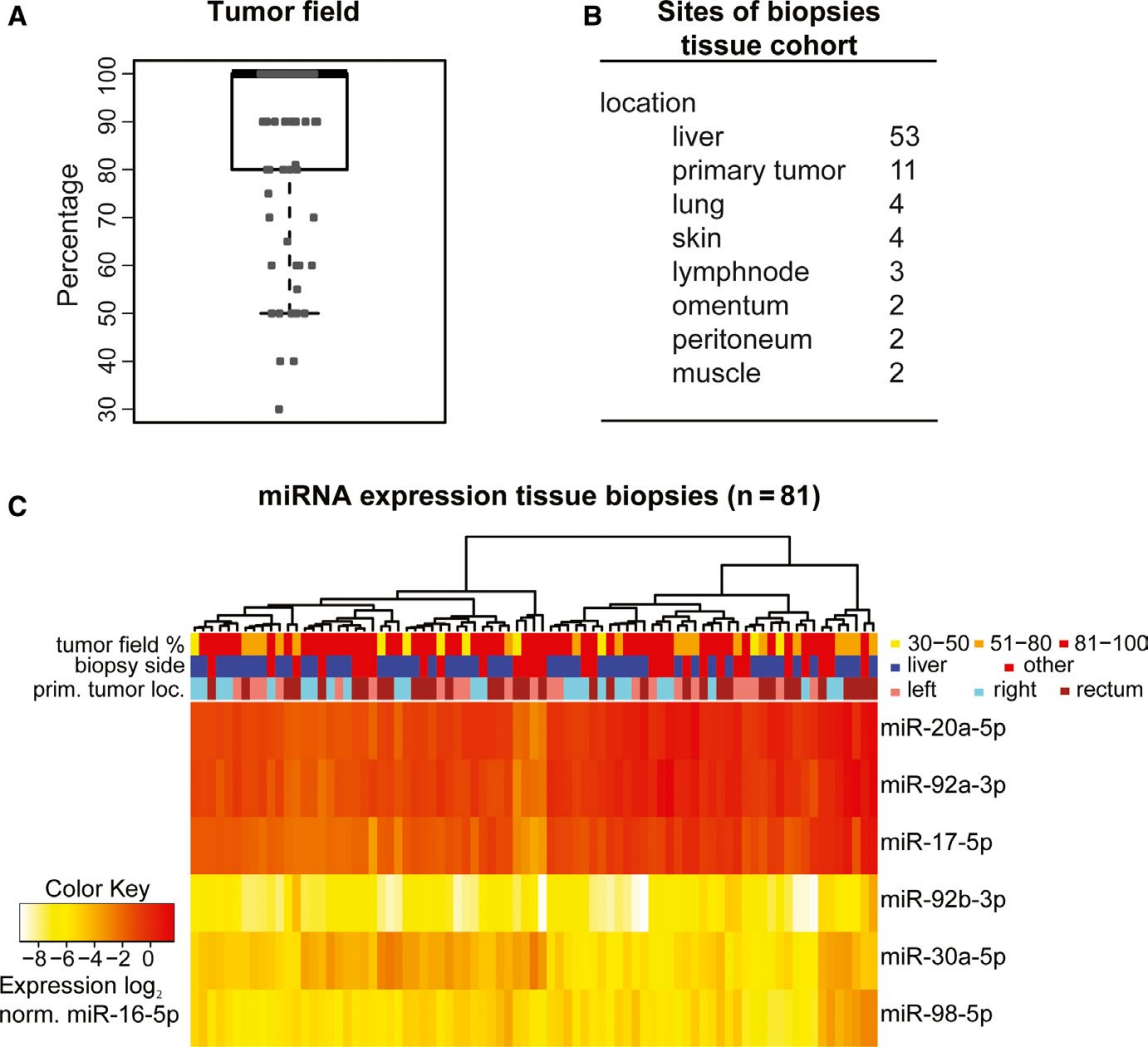
Tumor percentage, sample characteristics, and tissue miRNA expression. A, Boxplot of percentage of tumor field (tumor stroma plus tumor cells) per biopsy. Tumor field percentage was 30%‐100% with a median of 100% and an average of 86%. B, numbers and location of the different biopsies included in the tissue cohort. C, Unsupervised cluster analysis of log_2_, normalized to miR‐16‐5p, tissue miRNA expression levels. prim. tumor loc.: primary tumor location; norm.: normalized to miR‐16‐5p

No significant differences were observed between the clinicopathological characteristics from the tissue cohort (N = 81) and the serum cohort (N = 93) (Table S1). Tumor field percentage ranged from 30% to 100% with a median of 100% and a mean of 86% (Figure 2A). Of the included samples in the tissue cohort 53 (65%) were from liver metastases. Biopsies were collected from eight different organs (Figure 2B). Unsupervised cluster analysis of log_2_ transformed, miR‐16‐5p normalized, tissue miRNA expression data revealed two specific clusters. These clusters were not related to tumor field percentage (categorized in 30‐50%, 51%‐80% and 81%‐100%), location of the primary tumor (left‐ vs right‐sided colon vs rectum), tumor‐site biopsy (liver vs other) (Figure 2C).

**FIGURE 3:**
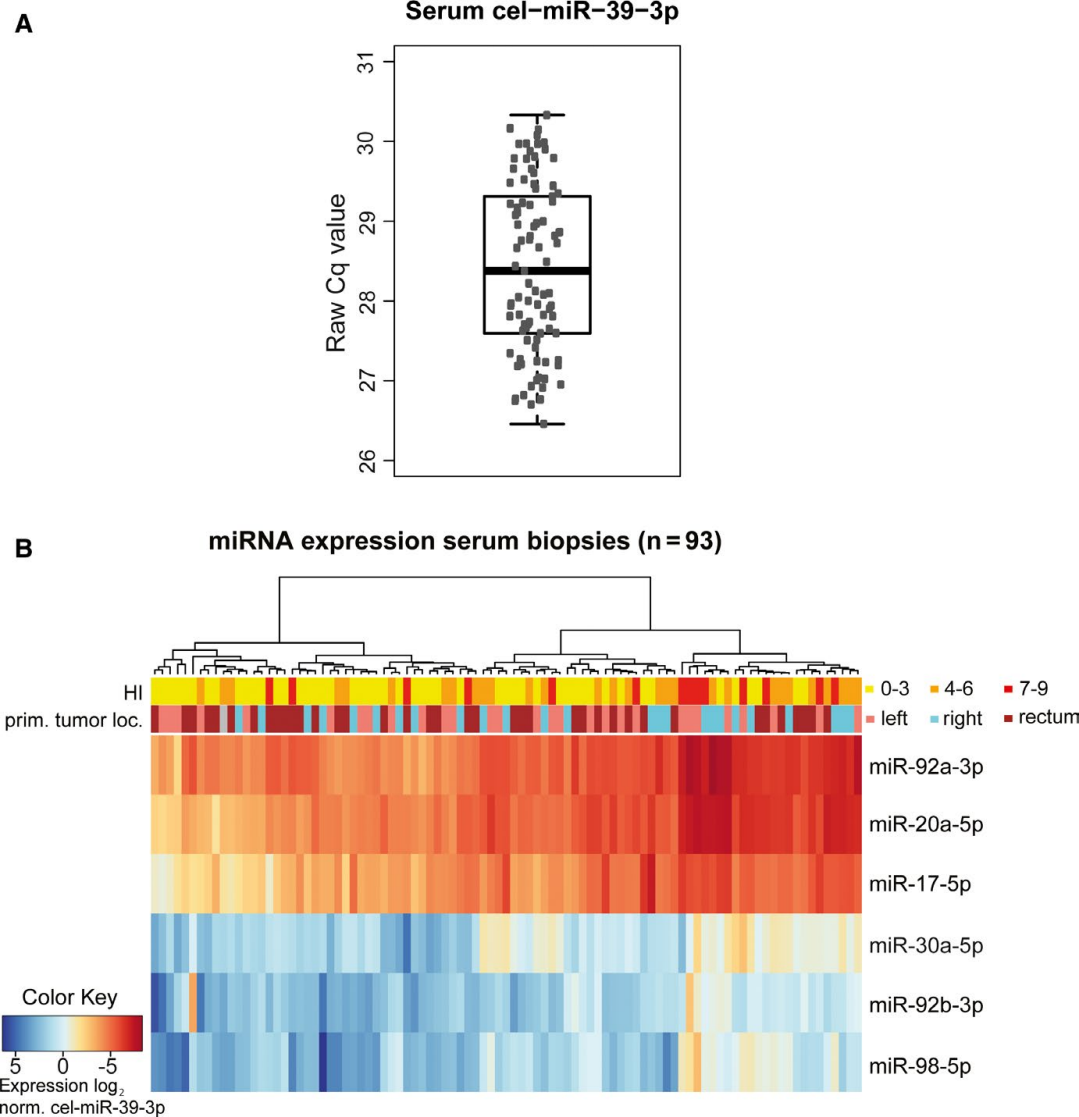
Serum RNA quality, sample characteristics, and serum miRNA expression. A, RNA quality control of RNA extracted from serum samples using cel‐miR‐39‐3p expression levels quantified with RT‐qPCR. Raw Cq values of cel‐miR‐39‐3p are presented in a boxplot. Raw Cq values were 26‐30 with a median of 28 B, Unsupervised cluster analysis of log_2_, normalized to cel‐miR‐39‐3p, serum miRNA expression levels. HI: Hemolytic index; prim. tumor loc.: primary tumor location; norm.: normalized to cel‐miR‐39‐3p

In the serum cohort, raw Cq values of cel‐miR‐39‐3p quantified with RT‐qPCR ranged from 26 to 30 with a median of 28,38 (Figure 3A). Unsupervised cluster analysis of log_2_, cel‐miR‐39‐3p normalized, serum miRNA expression data showed that cluster membership was not correlated to the location of the primary tumor, hemolytic index, or primary tumor location. In serum, high miRNA expression was observed for miR‐92a‐3p, miR‐20a‐5p, and miR‐17‐5p, whereas low expression was observed for miR‐30a‐5p, miR‐92b‐3p, and miR‐98‐5p (Figure 3B).

### 3.1 Prediction of treatment benefit

#### 3.1.1 Tissue

In the tissue cohort five patients (6.17%) showed PD, 39 (48.1%) had SD, and 37 (45.7%) had PR after three or four cycles of chemotherapy (Table 1). The miRNA expression levels from the fresh frozen cohort combined with the age, tumor differentiation, use of adjuvant chemotherapy after primary resection, and type of palliative systemic therapy revealed low predictive value for treatment response in contrast to the previously developed model. Here, using the previously developed predictive model for CR and PR vs PD that included miR‐92a‐3p, miR‐92b‐3p, and the three clinicopathological covariates as variables, resulted in an AUC of 0.60, 95% CI, 0.35‐0.85. Using the predictive model for CR and PR vs SD that included miR‐17‐5p, miR‐92a‐3p, miR‐92b‐3p, miR‐98‐5p, and differentiation grade of the primary tumor as variables, resulted in an AUC of 0.51, 95% CI, 0.37‐0.64.^15^

**TABLE 1:** Comparison of the patient cohort used to compute the initial response prediction models (original FFPE cohort) vs the cohort used for this study (FF cohort)

	Prior cohort FFPE	FF cohort	*P* value
	N = 81	N = 81	
Gender			.870
Female	30 (37.0%)	28 (34.6%)	
Male	51 (63.0%)	53 (65.4%)	
Age at diagnosis (median ‐ range)	61 (37 ‐ 81)	64 (28 ‐ 81)	.129
Primary tumor location:			
Rectal	20 (24.7%)	34 (42.0%)	.064
Left sided	30 (37.0%)	22 (27.2%)	
Right sided	31 (38.3%)	25 (30.9%)	
Stage			<.001
I	1 (1.20%)	0 (0.00%)	
II	13 (16.0%)	0 (0.00%)	
III	12 (14.8%)	0 (0.00%)	
IV	54 (66.7%)	81 (100%)	
Unknown	1 (1.20%)	0 (0.00%)	
Differentiation			.943
Well/moderately differentiated	54 (66.7%)	54 (66.7%)	
Poorly differentiated	15 (18.5%)	16 (19.8%)	
Other	0 (0.00%)	1 (1.23%)	
Unknown	12 (14.8%)	10 (12.3%)	
Prior adjuvant therapy			.072
Yes	7 (8.60%)	16 (19.8%)	
No	74 (91.4%)	65 (80.2%)	
Liver metastases only			<.001
No	57 (70.4%)	81 (100%)	
Yes	24 (29.6%)	0 (0.00%)	
LDH (cut‐off 250 ng/µL)			<.001
Elevated	55 (67.9%)	18 (22.2%)	
Normal	23 (28.4%)	56 (69.1%)	
Unknown	3 (3.70%)	7 (8.64%)	
CEA (cut‐off 5 ng/µL)			
Elevated	61 (75.3%)	55 (67.9%)	.643
Normal	17 (21.0%)	22 (27.2%)	
Unknown	3 (3.70%)	4 (4.94%)	
First‐line treatment scheme			<.001
5‐FU monotherapy	14 (17.3%)	0 (0.00%)	
Oxaliplatin‐based regimen	57 (70.4%)	81 (100%)	
Irinotecan‐based regimen	10 (12.3%)	0 (0.00%)	
Use of first‐line Bevacizumab			<.001
No	51 (63.0%)	21 (25.9%)	
Yes	30 (37.0%)	60 (74.1%)	
Best response to first‐line treatment^a^			
Complete response (CR)	2 (2.5%)	0 (0.00%)	.025
Partial response (PR)	36 (44.4%)	37 (45.7%)	
Stable disease (SD)	28 (34.6%)	39 (48.1%)	
Progressive disease (PD)	15 (18.5%)	5 (6.17%)	
Tissue specimen			<.001
Primary tumor	70 (86.4%)	10 (13.5%)	
Metastasis	11 (13.6%)	64 (86.5%)	

Note

Data from the original FFPE cohort were adapted from Neerincx et al.^15^

Abbreviations: CEA, carcinoembryonic antigen; FF, fresh frozen; FFPE, Formalin‐fixed paraffin‐embedded; LDH, lactate dehydrogenase.

^a^ According to RECIST 1.1. For the original FFPE cohort best response to first‐line treatment was used as clinical endpoint, while for the FF cohort response after three to four cycles of chemotherapy was used as clinical endpoint in this table.

**FIGURE 4:**
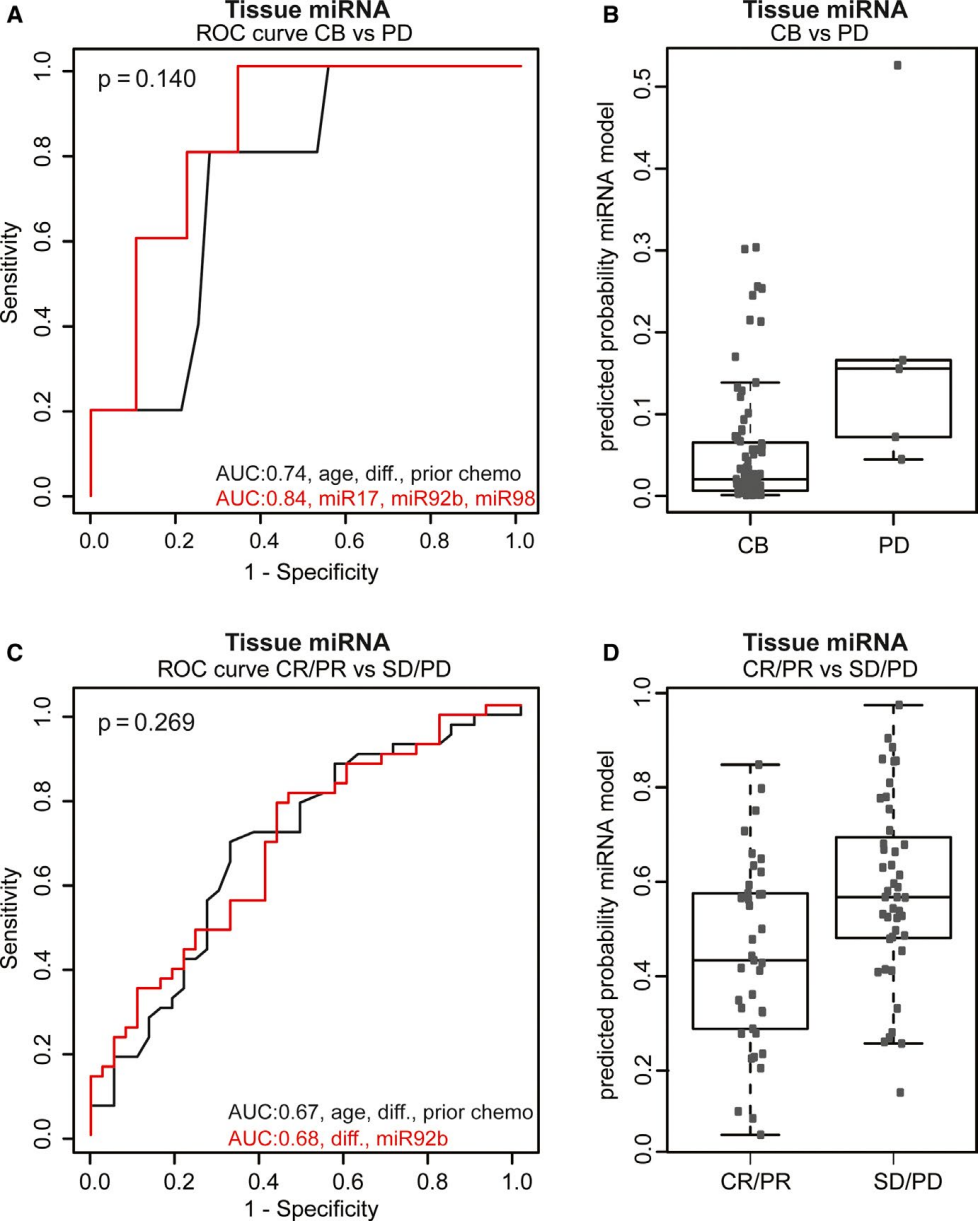
Performance of the predictive models in the tissue cohort. A, ROC curve for clinical benefit (n = 76) vs progressive disease (n = 5) after three to four cycles of chemotherapy. Black line: the model with the clinicopathological factors: age, differentiation and prior use of chemotherapy. Red line: the final model after backward selection using the clinicopathological factors combined with miRNA expression levels of miR‐17‐5p, miR‐92b‐3p, and miR‐98‐5p. B, The predictive probabilities for CB vs PD calculated with the miRNA model presented with the red line in (A) (clinicopathological factors combined with miR‐17‐5p, miR‐92b‐3p, and miR‐98‐5p). The median predicted probability of PD and CB is both 0.16. C, ROC curve for objective response (CR and PR (n = 37)) vs SD and PD (n = 44) after three to four cycles of chemotherapy. D, The predictive probabilities for CR and PR vs SD and PD calculated with the miRNA model presented with the red line in (C) (differentiation, miR‐92b‐3p). Median predicted probabilities are 0.47 and 0.58 for CR/PR and SD/PD, respectively. AUC, area under the curve; CB, clinical benefit; diff., differentiation; CR, complete response; PD, progressive disease; PR, partial response; ROC, receiver operator characteristics; SD, stable disease

When performing a new backward selection starting with all nine variables and using AIC for model selection, the best predictive model contained expression levels of miR‐17‐5p, miR‐92b‐3p, and miR‐98‐5p only. Prediction of clinical benefit vs progressive disease at first evaluation by measured expression levels of these three miRNAs revealed a model with an AUC of 0.85 (95% CI: 0.72‐0.91) (Figure 4A). The AUC of this model is higher compared to the AUC of the model with the parameters age, tumor differentiation, and prior use of adjuvant chemotherapy alone (AUC: 0.74, 95% CI, 0.56‐0.92), but did not reach statistical significance (*P* = .14). The calculated predicted probabilities from the model are visualized in Figure 4B. For PD the predicted probabilities ranged between 0.04 and 0.53 with a median of 0.16 and for CR, PR and SD the predicted probabilities ranged between < 0.01 and 0.30 with a median of 0.16 (Figure 4B). Addition of miRNA expression levels to the clinicopathological covariates did not significantly improve the performance of the model to discriminate between patients with objective response (CR and PR) (n = 37) vs SD and PD (n = 44), (AUC: 0.67, 95% CI:0.55‐0.79 vs AUC: 0.70, 95% CI:0.56‐0.80 with miRNAs, *P* = .27) (Figure 4C), with a wide range of predicted probabilities (Figure 4D). For objective response the predicted probabilities ranged between 0.17 and 0.81 with a median of 0.47 and for SD and PD this ranged between 0.25 and 0.91 with a median of 0.58 (Figure 4D).

#### 3.1.2 Serum

**FIGURE 5:**
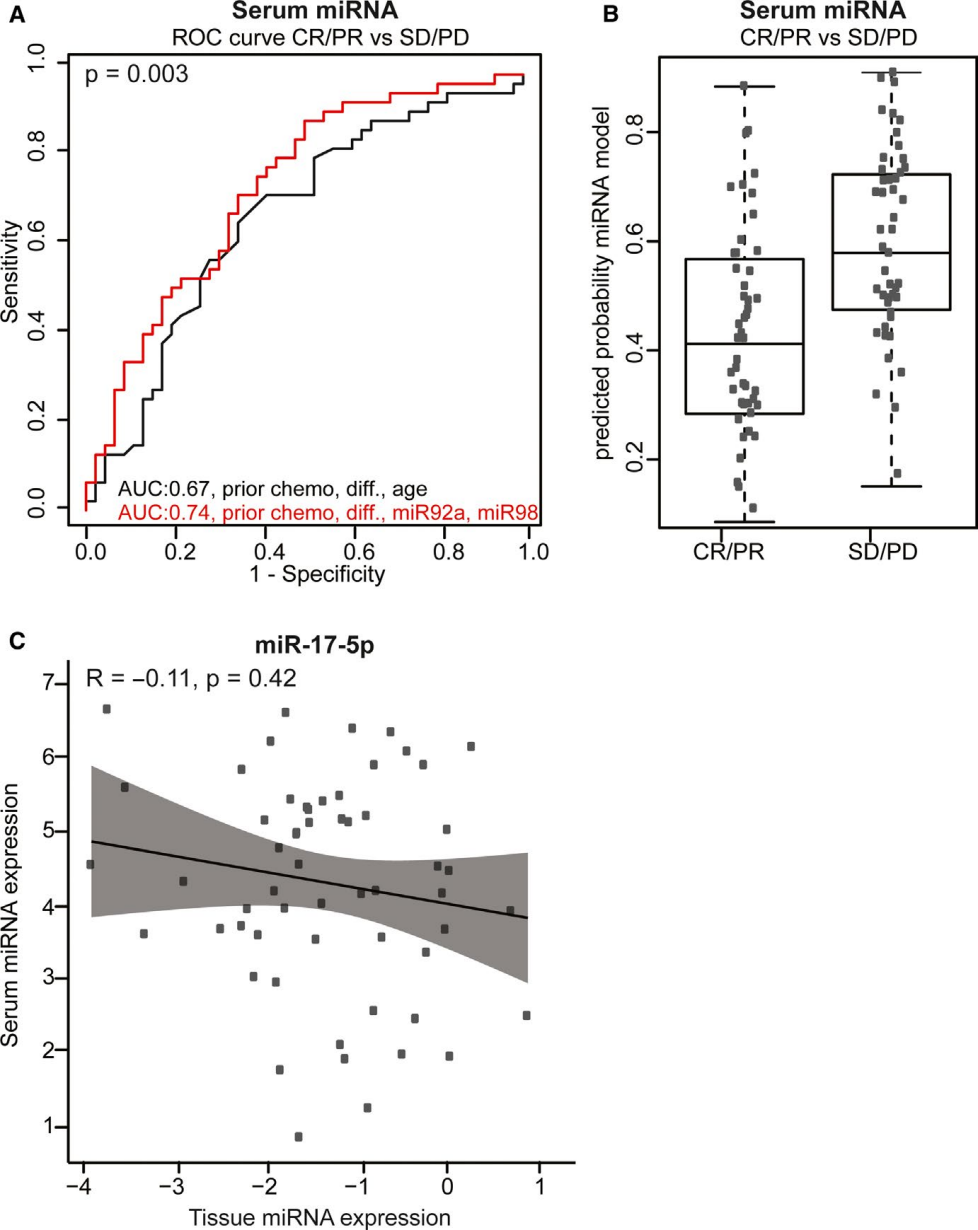
Performance of the predictive models in the serum cohort. A, ROC curve analysis for prediction of CR/PR (n = 46) vs SD/PD (n = 47) after three to four cycles of chemotherapy in the serum cohort. Black line: the model with the clinicopathological factors: age, differentiation and prior use of chemotherapy. Red line: the final model after backward selection using the clinicopathological factors combined with expression levels of miR‐92a‐3p and miR‐98‐5p. B, Individual predicted probabilities of the model with miRNAs presented with the red line in (A) for CR/PR vs SD/PD after three to four cycles of chemotherapy (prior chemo, differentiation, miR‐92a‐3p and miR‐98‐5p). Median predicted probability for CR/PR and SD/PD was 0.42 and 0.59, respectively. C, Correlation plot of log_2_ normalized tissue miRNA expression levels (*x*‐axis) and log_2_ normalized serum miRNA expression levels (*y*‐axis). Data are presented for miR‐17‐5p only, similar results were observed for other miRNAs (data not shown). AUC, area under the curve; CR, complete response; diff., differentiation; PD, progressive disease; PR, partial response; ROC, receiver operator characteristics; SD, stable disease.

In the serum cohort, logistic regression analysis could not be performed because only three (3.2%) patients showed PD after three to four cycles of chemotherapy. When separating the study cohort in objective response vs SD and PD after three to four cycles of chemotherapy, a model with miR‐92a‐3p and miR‐98‐5p expression combined with previous adjuvant chemotherapy and tumor differentiation showed a significant better performance compared to the model with clinicopathological factors alone (AUC 0.74, 95%CI 0.64‐0.84 vs AUC 0.67, 95%CI 0.56‐0.78; *P* = .003) (Figure 5A). The calculated predicted probabilities are visualized in Figure 5B which ranged between 0.08 and 0.88 with a median of 0.42 for CR and PR (n = 46) vs 0.15 and 0.90 with a median of 0.59 for SD and PD (n = 47) (Figure 5B). From 58 patients, tissue and serum expression data were available. There was no correlation between tissue and serum expression of these six miRNAs (*R* = −0.11; *P* = .42) (Figure 5C).

### 3.2 Comparison with original formalin‐fixed paraffin‐embedded (FFPE) cohort

Baseline characteristics of current FF cohort and the original FFPE cohort are shown in Table 1. There are significant differences in tumor stage (*P* < .001), with the current FF cohort including only patients with extrahepatic disease (stage IV) at diagnosis vs all stages at diagnosis in our original cohort. In the current FF cohort, a lower percentage of elevated LDH (*P* < .001) was observed and baseline tissue biopsies were from different locations (metastasis vs primary tumor) (*P* < .001). Systemic treatment schedules differed significantly for both chemotherapy (*P* < .001) and use of bevacizumab (*P* < .001). Also, response to therapy was significant different (*P* = .03) Table 1.

**FIGURE 6:**
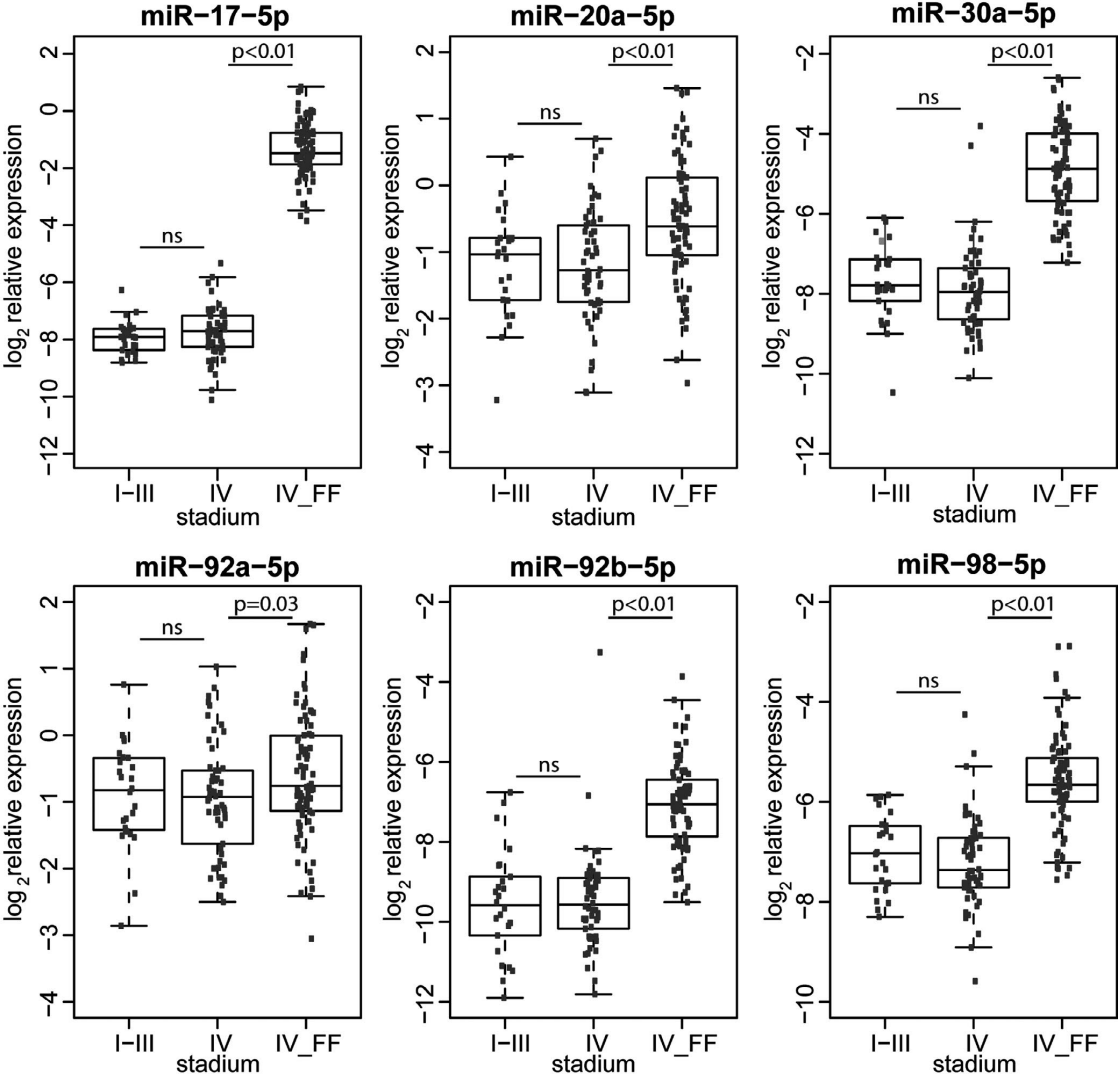
miRNA expression in the original FFPE cohort compared to the fresh frozen cohort. The original FFPE cohort was divided in stage I‐III and IV. The cohort consisted only of patients with stage IV disease (IV_FF). Differences in expression were compared between stadium I‐III (n = 26) and IV (n = 54) from the original FFPE cohort, and between stadium IV (n = 54) from the original FFPE cohort and IV_FF (n = 81) from the fresh frozen cohort using a two‐tailed unpaired *t* test. Log_2_ expression levels are presented as average from duplicate measurements relative to miR‐16‐5p. miRNA data were adapted from Neerincx et al.^15^ NS: not significant. FF, fresh frozen; FFPE, formalin‐fixed paraffin‐embedded; IV_FF, stadium IV fresh frozen cohort

No differences were observed between miRNA expression in stage I‐III and IV in the original FFPE cohort. All six miRNAs were significantly higher expressed in the current FF cohort compared to the original FFPE cohort ((*P* = .03; miR‐92a‐3p) and *P* < .01; miR‐17‐5p, miR‐20a‐5p, miR‐30a‐5p, miR‐92b‐3p, miR‐98‐5p) (Figure 6).

## 4 DISCUSSION

In this prospective study we aimed to validate the predictive value of a previously identified miRNA profile combined with clinicopathological factors in predicting clinical benefit from first‐line palliative systemic therapy in advanced mCRC.^15^ The same miRNA profile was quantified in serum specimens to test its predictive value as a liquid biomarker for clinical benefit to systemic treatment.

The predictive value of the tissue‐derived six miRNA profile combined with the clinicopathological factors could not be validated in patients with mCRC starting with first‐line systemic therapy. Compared to the cohort previously used for identification of the putative predictive profile, the clinical parameters of the currently analyzed FF cohort differed significantly with the most important difference being that this cohort consisted of only patients with stage IV disease. Studies have shown that expression levels of specific miRNAs differ between clinical tumor stages.^39^, ^40^ Although, for miR‐17, miR‐20a, miR‐30a, and miR‐92a previous studies have shown no significant relation between miRNA expression and stage.^20^, ^41^, ^42^ miR‐92b expression has not been related to stage before and Zhu et al found lower levels of miR‐98 expression in patients with stage III‐IV disease compared to patients with stage I‐II disease.^43^ Since there was no significant difference in miRNA expression between patients with stage I‐III (32%) vs stage IV (66.7%) disease in the previous cohort (see Table 1), we considered it worthwhile testing this signature in a cohort consisting of only patients with stage IV disease, who are starting first‐line palliative systemic therapy.

Additionally, in the original study cohort, patients were treated with different chemotherapeutic agents, that is, 5‐FU monotherapy, 5‐FU with oxaliplatin‐based regimens or 5‐FU with irinotecan‐based regimens, which was also an important covariate in the prediction model. In the current FF cohort, all patients received 5‐FU with oxaliplatin‐based chemotherapy and therefore this covariate consisted of one factor.

All six miRNAs were significantly higher expressed in the current FF cohort compared to the original FFPE cohort. Besides differences in patient characteristics, this could also be the result of methodological differences in RNA extraction and quantification, due to updated kits available from Qiagen (after Exiqon merged with Qiagen, the assays from Exiqon were updated by Qiagen to exhibit improved performance as stated in digital correspondence with Qiagen). In particular, the relatively low expressed miRNAs miR‐17‐5p, miR‐30a‐5p, miR‐92b‐3p, and miR‐98‐5p were significantly higher expressed in the updated assays.

Furthermore, the biopsy specimens in this cohort consisted of FF tissue, mainly from metastatic lesions, whereas in the original FFPE cohort biopsies consisted of FFPE tissue samples, mainly from the primary tumor. Although studies have shown good correlations between miRNAs quantified in paired FF and FFPE samples independent of the employed methodology,^5^, ^44^, ^45^, ^46^, ^47^ this factor cannot be completely ruled out since an analysis of FF vs FFPE of paired samples using similar methods has not been performed for this study.

We previously demonstrated that miRNA profiles of primary tumor and metastases are similar, making the biopsy site an unlikely explanation for differences in miRNA expression levels.^16^ The miRNAs selected for this study were based on next generation sequencing (NGS) results from FF tissue of 88 patients with advanced CRC as described previously by Neerincx et al.^15^ The initial clinical endpoint in that study was clinical benefit vs progressive disease defined as best response to first‐line treatment using Response Evaluation Criteria in Solid Tumors (RECIST version 1.1).^29^ In the study presented here, the response was assessed at first evaluation after three to four cycles of systemic therapy. In this cohort, only five patients had progressive disease at first evaluation (6.8%). Although this is a low number for predictive modeling, the miRNAs and clinicopathological parameters selected for this study are based on two independent cohorts of a total of 169 patients.^15^ The patient cohort in the current study was used for further validation of the miRNAs and clinicopathological parameters. If these parameters were truly predictive for response to chemotherapy a clinically relevant model should identify these five patients as nonresponders.

Using other evaluation time points (ie, after six to eight cycles, progression‐free survival 6 or 9 months) did not improve the predictive value either.

miRNA expression by tumor cells may affect serum levels. This depends on the balance between the expression levels of tumor cells and the endogenous production of normal (circulating blood) cells in the body. High miRNA expression and release by tumor cells and low endogenous production are ideal for an optimal predictive biomarker. Here, three of six miRNAs also originate from blood cells (miR‐17‐5p, miR‐92a‐3p, and miR‐20a‐5p) and therefore have high baseline serum expression levels.^32^, ^48^ This was confirmed in the current study. Consequently, miRNAs released from tumors may be insufficient to significantly alter these high baseline levels. On the contrary, miR‐30a‐5p, miR‐92b‐3p, and miR‐98‐5p were proven to have low baseline serum expression levels, but are also expressed at low levels in CRC tissue. Again, these low miRNA levels released from tumors might be insufficient to allow detection (with current technology) of alterations in baseline serum expression levels.

Nevertheless, previous studies have identified a predictive and/or prognostic value in CRC of serum expression levels of four (miR‐17‐5p, miR‐20a‐5p, miR‐30a‐5p, miR‐92a‐3p) of the six miRNAs selected for this study.^49^, ^50^, ^51^ Probably other factors, for example the composition of the tumor microenvironment, the percentage of immune cell infiltration or circulating inflammatory indicators, which are also important factors for therapy response, may alter ci‐miRNA levels as well. In our dataset the addition of miR‐92a‐3p and miR‐98‐5p significantly improved the AUC of the ROC curve for predicting response to chemotherapy. However, as demonstrated by the wide the range of calculated predicted probabilities in Figure 5B, the predictive value remains moderate and as such not useful in clinical practice.

To reduce background noise of endogenous miRNAs in blood cells, miRNAs could be quantified in exosomes. It is hypothesized that exosomes released from tumor cells are enriched with a tumor specific miRNA signature.^52^

The predictive models generated from our previous cohort could not be validated in the current dataset. In the adjusted models for the current FF cohort, serum miR‐98‐5p and miR‐92b‐3p levels improved response prediction compared to the clinicopathological factors alone. The other four miRNAs that were found predictive by others^49^, ^50^, ^51^ were excluded from the models because of insignificance and could not be validated in this cohort.

A different miRNA panel or replacing some miRNAs of the current signature could possibly lead to a more predictive model. However, the present study investigated the clinical value of our previously identified prediction model. The identification of novel predictive miRNAs for the use as clinical biomarker is beyond the scope of the current study and requires further investigation.

In conclusion, despite the known role of miRNAs in cancer biology and their favorable characteristics to serve as biomarkers for clinical benefit from systemic treatment, the predictive miRNA signature which was previously identified did not show significant strength to predict treatment benefit in the present sizeable cohort of patients with mCRC undergoing first‐line combination chemotherapy. This could be partly due to differences in patient population and the materials and methods used. It emphasizes the importance of thorough research on miRNAs as a predictive marker prior to using it in clinical decision‐making. Potential improvements, by selecting miRNAs in circulating vesicles may provide additional value in this matter and requires additional evaluation.

## CONFLICT OF INTEREST

The authors declare that they have no known competing financial interests or personal relationships that could have appeared to influence the work reported in this paper.

## AUTHOR CONTRIBUTIONS

Henk Verheul and Kees Verhoef were involved in principal investigator, conceptualization, investigation, funding acquisition, writing revisions and supervision. Dennis Poel, Elske Gootjes, Lotte Bakkerus, and Tineke Buffart were involved in conceptualization, formal analysis, investigation, methodology, project administration, writing original manuscript, statistical methods, data curation, and writing revisions. Dennis Poel was involved in visualization. Wim Trypsteen was involved in statistical analysis and methods. Nicole CT van Grieken and Hans J van der Vliet were involved in investigation. Henk Dekker was involved in methodology. All authors reviewed and edited the original article, and approved the final version.

## Supporting information

Fig S1

Table S1

Appendix S1

## Data Availability

Please contact the corresponding author for all data requested.
