# Supplementary figures and images for: A specific microRNA profile as predictive biomarker for systemic treatment in patients with metastatic colorectal cancer
Source: Cancer Med. 2020 Aug 30;9(20):7558–71. doi: 10.1002/cam4.3371 (PMC7571833; doi:10.1002/cam4.3371)

A

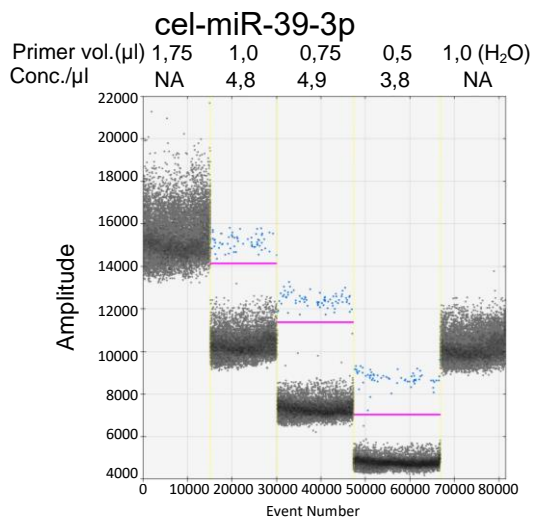

B

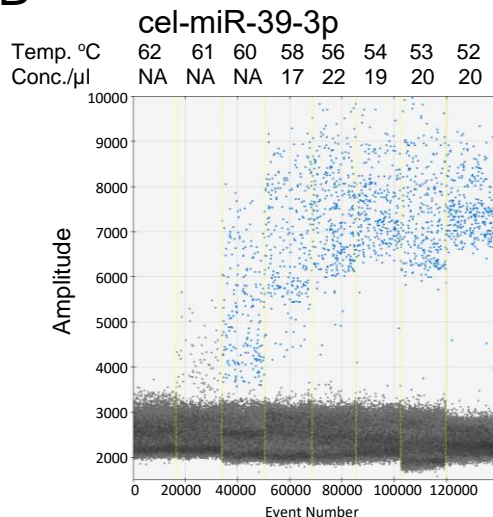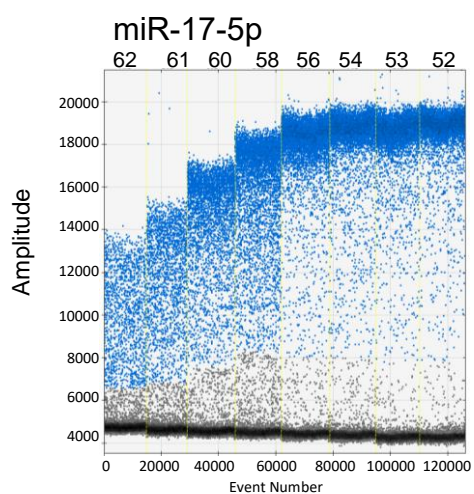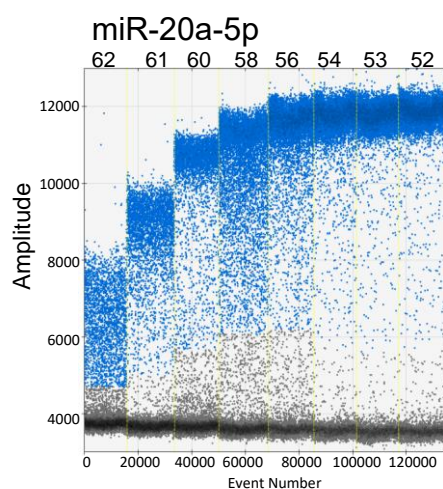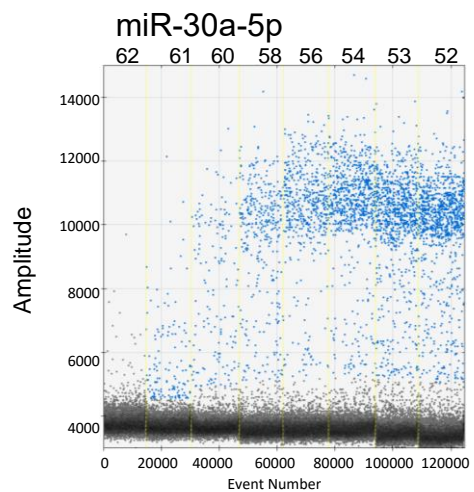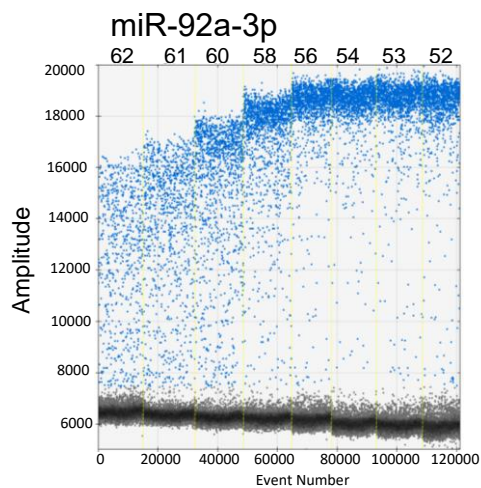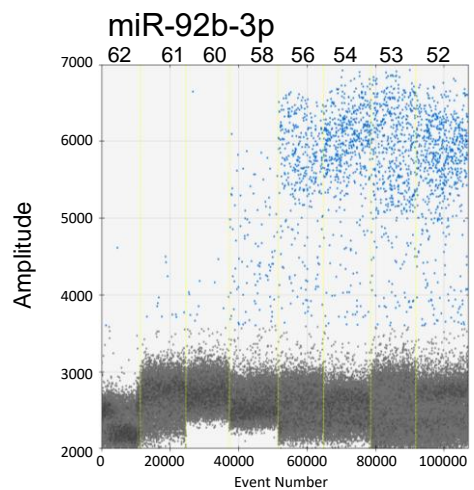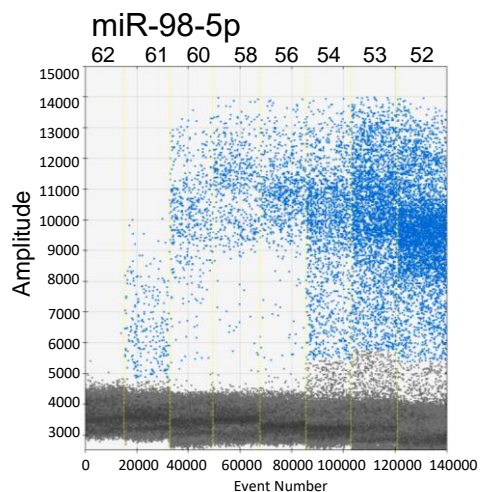

Supplement: Supplementary file 1 — Fig S1 [file CAM4-9-7558-s001.pdf]
