# Supplementary material for: A specific microRNA profile as predictive biomarker for systemic treatment in patients with metastatic colorectal cancer
Source: Cancer Med. 2020 Aug 30;9(20):7558–71. doi: 10.1002/cam4.3371 (PMC7571833; doi:10.1002/cam4.3371)
Supplement: Supplementary file 3 — Appendix S1 [file CAM4-9-7558-s003.pdf]

Supplementary appendix

Participating Centers and Principal Investigators of the ORCHESTRA study group.

Amphia Hospital, Breda, Dept. of Medical Oncology, Albert J Ten Tije

Isala, Zwolle, Dept. of Medical Oncology, Jan Willem B. de Groot

Radboud University Hospital, Nijmegen, Dept. of Medical Oncology, Sandra A. Radema

Meander Medical Center, Amersfoort, Dept. of Medical Oncology, Haiko J. Bloemendal

Noord West ziekenhuisgroep, Alkmaar, Dept. of Medical Oncology, Mathijs P. Hendriks

Hospital Amstelland, Amstelveen, Dept. of Medical Oncology, Annette A. v Zweeden

Fransiscus Gasthuis, Rotterdam, Dept. of Medical Oncology, Paul Hamberg

Haaglanden Medical Center, Den Haag, Dept. of Medical Oncology, Helgi H. Helgason

Bravis Hospital, Roosendaal, Dept. of Medical Oncology, Monique M. Troost

Elisabeth-TweeSteden hospital, Tilburg, Dept. of Medical Oncology, Laurens V. Beerepoot

Antoni van Leeuwenhoek, Amsterdam, Dept. of Medical Oncology, Cecile M.I. Grootscholten

Deventer hospital, Deventer, Dept. of Surgical Oncology, Hans Torrenga

Maasstad hospital, Rotterdam, Dept. of Medical Oncology, Brigitte C.M. Haberkorn

Jeroen Bosch hospital, Den Bosch, Dept. of Medical Oncology, Hans F.M. Pruijt

IJsselland hospital, Capelle aan de Ijssel, Dept. of Surgical Oncology, Maarten Vermaas

Amsterdam Medical Center, Amsterdam, Dept. of Surgical Oncology, Pieter J. Tanis

Admiraal de Ruyter hospital, Goes, Dept. of Medical Oncology, Henk van Halteren

Máxima Medical Center, Eindhoven, Dept. of Medical Oncology, Ard Vreugdenhil

Maastricht University Medical Center, Maastricht, Dept. of Medical Oncology, Rob L.H. Jansen

Sint Antonius hospital, Nieuwegein, Dept. of Medical Oncology, Maartje Los

Medical Center Leeuwarden, Leeuwarden, Dept. of Medical Oncology, Marco B. Polée

Albert Schweitzer hospital, Dordrecht, Dept. of Medical Oncology, Marija Trajkovic-Vidakovic

Dijklander hospital, Hoorn, Dept. of Medical Oncology, Edwin van Breugel

Gelre hospitals, Apeldoorn, Dept. of Medical Oncology, Hielke Meulenbeld

Onze Lieve Vrouwe Gasthuis, Amsterdam, Dept. of Medical Oncology, Jetske M. Meerum Terwogt
